# Shape Tuning and Size Prediction of Millimeter-Scale Calcium-Alginate Capsules with Aqueous Core

**DOI:** 10.3390/polym12030688

**Published:** 2020-03-19

**Authors:** Jinchao Zhao, Qing Guo, Wei Huang, Teng Zhang, Jing Wang, Yu Zhang, Leping Huang, Youhong Tang

**Affiliations:** 1 Hubei Biomass Fibers and Eco-dyeing & Finishing Key Laboratory, School of Chemistry and Chemical Engineering, Wuhan Textile University, Wuhan 430200, China; jczhao@wtu.edu.cn (J.Z.); whiteguo0524@163.com (Q.G.); huangwei19950904@163.com (W.H.); jing0123wang@163.com (J.W.); 2 School of Material Science and Engineering, Wuhan Textile University, Wuhan 430200, China; 1715043028@mail.wtu.edu.cn (T.Z.); zhangyuer126@126.com (Y.Z.); 3 Institute for NanoScale Science & Technology, College of Science and Engineering, Flinders University, South Australia 5042, Australia

**Keywords:** calcium-alginate capsules, size, sphericity, mechanical stability, drug release

## Abstract

Controllable feature and size, good mechanical stability and intelligent release behavior is the capsule products relentless pursuit of the goal. In addition, to illustrate the quantitative relationship of structure and performance is also important for encapsulation technology development. In this study, the sphericity and size of millimeter-scale calcium sodium alginate capsules (mm-CaSA-Caps) with aqueous core were well tuned by manipulating the viscosity, surface tension, and density of CaCl_2_/carboxyl methyl cellulose (CMC) drops and sodium alginate (SA) solution. The well-tuned mm-CaSA-Caps showed significant mechanical and control-releasing property effects. The results showed that the prepared mm-CaSA-Caps were highly monodispersed with average diameter from 3.8 to 4.8 mm. The viscosity of the SA solution and the viscosity and surface tension of the CaCl_2_/CMC solution had significant effects on the mm-CaSA-Caps sphericity. Uniform and spherical mm-CaSA-Caps could be formed with high viscosity CaCl_2_/CMC solution (between 168.5 and 917.5 mPa·s), low viscosity SA solution (between 16.2 and 72.0 mPa·s) and decreased surface tension SA solution (by adding 0.01 wt.% poloxamer 407). The diameter of the mm-CaSA-Caps could be predicted by a modified Tate’s law, which correlated well with the experimental data. The Caps with sphericity factor (SF) < 0.07 had better mechanical stability, with the crushing force 2.91–15.5 times and the surface Young’s modulus 2.1–3.99 times higher than those of the non-spherical Caps (SF > 0.07). Meanwhile, the spherical Caps had a more even permeation rate, which was helpful in producing uniform and sustained releasing applications in foodstuff, medicine, agriculture and chemical industry.

## 1. Introduction

Fish-egg-like calcium sodium alginate (CaSA) matrix capsules (Caps) with an aqueous core at the millimeter scale prepared using the extrusion dripping method have been studied in food [1], agriculture [2] and biomedical industries [3,4]. Sodium alginate (SA), an anionic linear polysaccharide of 1,4-linked-α-L-guluronic acid and β-D-mannuronic acid residues that are widespread in seaweeds, can produce gel in the presence of divalent and trivalent cations and form non-toxic and biocompatible mini-dose containers by dripping SA into a calcium salt coagulation bath under very mild crosslinking conditions [5]. The active compounds, from water to oil, encapsulated in the CaSA hydrogel matrix can be protected by a semipermeable gel membrane with controlled release and shielding functions [6,7]. For core-shell structures, CaCl_2_ droplets containing the active molecule and non-gelling polymer used to modulate viscosity and density are dropped into a SA bath to form the gel membrane in the interface of alginate molecules and calcium cations [8]. This inverted extrusion dripping method is popular due to its simple setup. As well, it ensures that bioactive materials to be encapsulated never come into contact with the ionotropic gel-forming polymers and remain within their original environment [8]. Moreover, this method is more likely to obtain microcapsules within a narrow dimensional range and with high encapsulation efficiency, thereby proving a good choice for industrialized production [9].

Sphericity of diameter and mono-dispersion are required for the production of high-quality mm-CaSA-Caps. It has been reported that capsules are easily deformed during the dripping process [5,10]. In the production of traditional CaSA solid core beads with a desired size and spherical shape [8], SA solution is dripped into a Ca^2+^ coagulation bath and some trial-and-error attempts are often required with regard to aspects of the liquid formulation and experimental setup, e.g., solution viscosity or surface tension, tip size and collecting distance, aspects that are important for mm-CaSA-Caps preparation. Lee et al. [11] discussed the influence of process variables of the method on the capsule size and shape by optimizing preparation conditions, notably the concentration of SA and CaCl_2_, gelation time, dripping tip diameter, gelation solution height and stirring rate of the gelation bath. Messaoud et al. [12] reported that the inner polymer thickening agent had an impact on membrane stability and could act as an internal coating or provide mechanical reinforcement that could effectively prevent breakage and leakage of capsules.

Efficiency of mass transfer and mechanical stability are vital for capsule applications. The capsules should not only control sustained release but also protect the inner fluid from mechanical stresses [13], especially with regard to their biological function. For example, red blood cells, which are natural microcapsules, can squeeze through capillaries with only 10% of their diameter without any damage due to their lowest bending resistance membranes [14]. Researchers have kept the focus on membrane modification to prepare capsules with specified releasing and mechanical properties that can meet different needs in various environments [15,16,17,18,19,20,21], but few researchers have paid attention to capsule shape and size manipulation and their effects on the final properties.

In this work, carboxyl methyl cellulose (CMC) was added as thickener to CaCl_2_ solution and dripped into a SA bath to form mm-CaSA-Caps. The diameter and sphericity of the capsules was regulated by controlling viscosity, surface tension and density of SA and CaCl_2_/CMC solutions. Capsule diameter was predicted based on the modified Tate’s law. In particular, the sphericity factor (SF) effect on the mechanical properties and permeability of mm-CaSA-Caps is discussed.

## 2. Experimental

### 2.1. Materials

Sodium alginate (SA, 10 g/L, 20 °C, > 0.02 Pa·s) was dissolved in deionized water as a gelation bath. Sodium carboxymethyl cellulose (CMC, 20 g/L, 25 °C, 800–1200 mPa·s) was used as a non-gelling thickener agent to increase the viscosity of the aqueous core solution. Tween 80, a nonionic surfactant, was used to modify the surface tension of the CMC solutions. Dextrose monohydrate, glycerin and sodium hydroxide used were of analytic grade. The above reagents were purchased from Sinopharm Chemical Reagent Co. Ltd., Shanghai, China. Poloxamer 407 (Lutrol F127, average Mn~12600), a nonionic surfactant used to modify the surface tension of the SA solutions, was provided by BASF, Ludwigshafen, Germany. 3,5-dinitrosalicylic acid (98%) was purchased from Macklin, Shanghai, China. Deionized water was used throughout the experiments.

### 2.2. Preparation of mm-CaSA-Caps 

**Figure 1:**
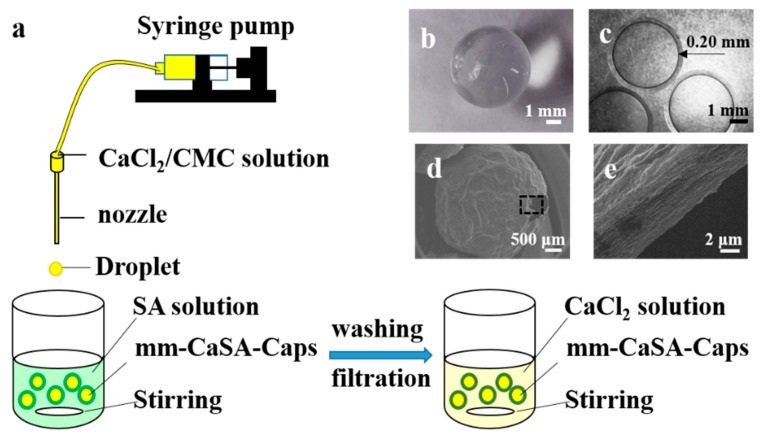
(**a**) Schematic drawing of the preparation process of millimeter-scale calcium sodium alginate capsules (mm-CaSA-Caps); (**b**) mm-CaSA-Caps (top view); (**c**) microscope image of the mm-CaSA-Caps (in water); (**d**) SEM image of the mm-CaSA-Caps (after freeze drying) and (**e**) high magnification of the rectangular part in (**d**).

The mm-CaSA-Caps were prepared at 20–25 °C by an extrusion dripping device [11] as shown in Figure 1a. In detail, a solution containing 1 wt.% CMC and 1.5 wt.% CaCl_2_ was loaded into a 20 mL plastic syringe, pumped dropwise with the flow rate of 0.5 mL/min by a dripping nozzle into a gelation bath containing 200 mL 0.5 wt.% SA solution. A hypodermic needle with the outer diameter of 1.06 mm (19G) was used as the dripping nozzle. The distance between the dripping nozzle and the surface of SA solution was fixed at 10 cm. After 10 min dripping process, the solution was stirred for 20 min, and the obtained capsules were then washed with deionized water three times. Then, an additional crosslinking process for the capsules was conducted for 15 min in 2.0 wt.% CaCl_2_ solution. Finally, the mm-CaSA-Caps were stored in deionized water before measurement. As shown in Figure 1, the mm-CaSA-Caps with the diameter of 4.170 ± 0.072 mm have a beautiful round shape (Figure 1b). The transparent membrane of the capsules was formed of an even 0.20 mm film (Figure 1c). After freeze drying for 24 h, the mm-CaSA-Caps retained their spherical structure (Figure 1d). The cross section of the membrane (Figure 1e) had the typical layered porous structure of a common hydrogel after dehydration, as shown in the high magnification (Figure 1e) of a rectangular portion from Figure 1d.

### 2.3. Experimentation

The viscosity and density of SA and CaCl_2_/CMC solutions were adjusted by concentration of solution. The concentrations of CMC were 0.5, 0.75, 1.0, 1.25 and 1.5 wt.%. The concentrations of SA were 0.25, 0.5, 0.75, 1.0 and 1.25 wt.%. The surface tension of the SA solution was adjusted by adding poloxamer 407. The concentrations of poloxamer 407 were 0, 0.01, 0.05, 0.1 and 0.2 wt.%. The surface tension of the CMC/CaCl_2_ solution was adjusted by adding Tween 80. The concentrations of Tween 80 were 0, 0.015, 0.025, 0.038, 0.075, 0.15 and 0.3 g/L.

### 2.4. Measurement of Solution Properties 

The viscosities of SA solution and CaCl_2_/CMC solution were determined by a rotary viscometer (NDJ-5S, Shanghai Pingxuan Scientific Instrument Co., Ltd., Shanghai, China) in accordance with ISO3219:1993. During the viscosity measurements, the temperature was kept at 25 °C. The data were the averages of 3 measurements. The surface tension was determined using a surface tension instrument (DCAT21, Dataphysics Instruments GmbH, Filderstadt, Germany) in accordance with ISO 304:1985. The data were the averages of 3 measurements. The density was measured using the pycnometer method in accordance with ISO 1675: 1985. The data were the averages of 3 measurements.

### 2.5. Capsule Characterization 

Images of the capsules in air and water were captured by a digital camera (BL-SM500, Jinhua Oseelang Trade Co., Ltd., Jinhua, China). The diameter of the capsules (*D*, mm, using *D_min_* as the diameter *D*.) was measured with a digital micrometer and the shape of the capsules was quantified using the sphericity factor (*SF*) in Equation (1) [22,23]

*SF* = (*D_max_* − *D_min_*)/(*D_max_* + *D_min_*) (1)

where *D_max_* is the longest f diameter length, and *D_min_* is the shortest diameter length perpendicular to *D_max_*. The capsules were considered spherical when *SF* < 0.07. To determine *D* and *SF*, 15 capsules for each condition were randomly chosen.

No apparent trends of capsule membrane thickness were observed when the CMC concentration was increased [11]. Therefore, the effect of SF on the mechanical and diffusion properties of the capsules was investigated with the CMC concentration increased. Mechanical properties of the capsules were measured with a transducer rotational rheometer (Ar2000ex, TA Instruments, New Castle, DE, USA) and the force gap test was used to compress the capsules from 4.2 to 0.05 mm with the linear compression speed of 0.3 mm/s [12]. At least five replications were considered for each capsule type. The surface Young’s modulus (*E_s_*), the measure of mm-CaSA-Caps stiffness, was estimated by analyzing the force–displacement curves in the range of small deformations and using Equation (2) [24]:

(2) $$F=\frac{4E_{S}h}{r\sqrt{3\left(1-\nu_{s}^{2}\right)}}d_{D}$$

where *d_D_* is the Caps displacement, *F* is the measured force, *h* is the membrane thickness, which is imported as a fixed value of 0.20 mm, *r* is the radius of the Caps and *υ_s_* is the surface Poisson ratio for which a value of 1/2 was assumed for the alginate hydrogel [25].

The permeability of mm-CaSA-Caps was measured by the diffusion of glucose from bulk solution into intra-hollow Caps. The concentration of glucose was measured by the dinitrosalicylic colorimetric method (DNS) [26]. In detail, 500 mm-CaSA-Caps was added into 100 mL glucose solution (20 mg/mL), of which 1 mL was taken at intervals into a 10 mL glass tube, followed by 1.5 mL DNS reagent and 1.5 mL deionized water, all shaken up and boiled for 5 min, cooled to room temperature, brought to volume in a 10 mL volumetric flask, and tested at 540 nm by an ultraviolet spectrophotometer. The variation of glucose concentration with time was ascertained against the glucose standard curve.

### 2.6. Prediction of the Diameter of mm-CaSA-Caps

The diameters of CaSA capsules (*d_p_*, mm) could be predicted using the modified Tate’s law mathematical model, given as Equation (3):

(3) $$d_{p}{=K(6d}_{t}{\gamma /\rho g)}^{\frac{1}{3}}$$

where *K* is the overall size correction factor, *d_t_* is outer diameter of the needle (mm), *γ* is the surface tension of CaCl_2_/CMC solution (mN/m), *ρ* is the density of CaCl_2_/CMC solution (kg/m^3^) and g is the gravitational force (9.81 m/s^2^). Compared to the Tate’s law, the overall size correction factor (*K*) was introduced into the modified model to take into account the residual liquid at the dripping nozzle (*K_LF_*) and the change of droplet diameter after gelation (*K_SF_*). The *K_LF_* and *K_SF_* were calculated by Equations (4) and (5) [27].

(4) $$K_{LF}=0.98-0.04d_{t}$$

(5) $$K_{SF}{=D/d}_{d}$$

The outer diameter of the needle was 1.06 mm and *K_LF_* was 0.9376 in this study. The diameter of the droplet (*d_d_*, mm) was calculated by Equation (6) [27]:

(6) $$d_{d}=\sqrt[{3}]{\frac{6V}{\pi }}=\sqrt[{3}]{\frac{6m}{\pi \rho }}$$

where *V* is the volume of CaCl_2_/CMC solution droplet (m^3^), *m* is the mass of the CaCl_2_/CMC solution droplet (kg) and *ρ* is the density of CaCl_2_/CMC solution (kg/m^3^). In this equation, it is assumed that the droplet is in the form of a sphere.

To evaluate the accuracy and reliability of the capsule diameter prediction model, average absolute deviation (AAD) and maximum absolute deviation (MAD) were introduced. AAD analysis indicates the average deviation of the experimental data and MAD analysis reflects the degree of deviation of the experimental data. AAD is given by Equation (7) [27] and MAD is defined as the maximum value of the absolute deviation between the experimental data and the reference values.

(7) $$\mathrm{AAD}=\overset{n}{\underset{i=1}{\sum }}\left|\frac{d_{p}-D}{d_{p}}\right|\times \frac{100}{n}$$

where *n* is sample number of the capsules.

## 3. Results and Discussion

### 3.1. Solution Properties 

**Figure 2:**
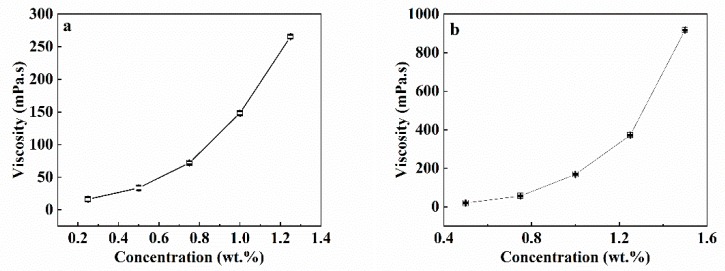
Viscosity of the solution as the function of SA (**a**) and CaCl_2_/CMC (**b**) concentration.

The viscosities of the SA and CaCl_2_/CMC solutions are shown in Figure 2. The viscosity of the SA solution increased from 16.2 to 516.0 mPa·s in an exponential manner with the SA concentration increasing from 0.25 to 1.25 wt.% (Figure 2a). These results were in good agreement with previous studies [9,28]. Similar trends were also evident in the CaCl_2_/CMC solution. The viscosity of the CaCl_2_/CMC solution increased from 20.0 to 917.5 mPa·s as the concentration of CaCl_2_/CMC increased from 0.5 to 1.5 wt.% (Figure 2b).

**Figure 3:**
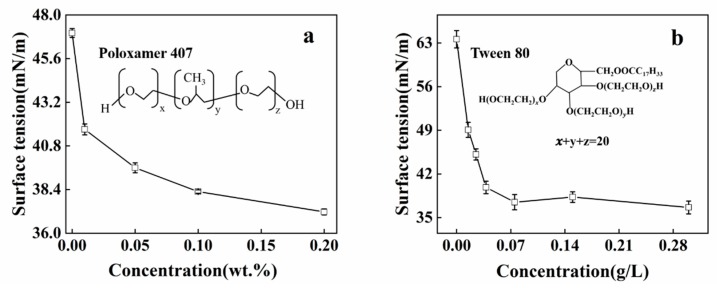
The surface tension of the solution as the function of (**a**) 0.5 wt.% SA solution with different concentrations of poloxamer 407 and (**b**) 1.5 wt.% CaCl_2_ and 1 wt.% CMC solution with different Tween 80 concentrations. Inserts show the chemical structures of poloxamer 407 and Tween 80.

Two surfactants were used, poloxamer 407 and Tween 80, which reduced the surface tension of the SA and CMC solutions respectively. The critical micelle concentration of Tween 80 is 0.014 g/L, according to literature data [29]. As 0.1 wt.% poloxamer 407 was added into the SA solution, the surface tension decreased from 47.01 ± 0.246 to 41.71 ± 0.401 mN/m, as shown in Figure 3a. The surface tension of the SA/poloxamer continued to decrease when the concentration of poloxamer 407 increased. As shown in Figure 3b, the surface tension of the CaCl_2_/CMC solution also obviously decreased with the addition of Tween 80. When 0.015 g/L Tween 80 was added into the CaCl_2_/CMC solution, the surface tension of the mixed solutions decreased from 63.41 to 49.01. The surface tension continued to decrease with the increase in Tween 80 content, until it was close to a constant 37 mN/m at 0.075 g/L Tween 80. Comparison of Figure 3a,b shows that the surface tension of the SA solution with poloxamer 407 decreased gradually, unlike the obvious critical micelle concentration appearing in the CaCl_2_/CMC/Tween 80 solution. Compared to the behavior of low-molecular-weight nonionic surfactants, the aggregation behavior of PEO-PPO-PEO surfactants like poloxamer 407 is complex, in that aggregation of poloxamer 407 occurs over a range of concentrations rather than at a unique critical micelle concentration [30].

**Figure 4:**
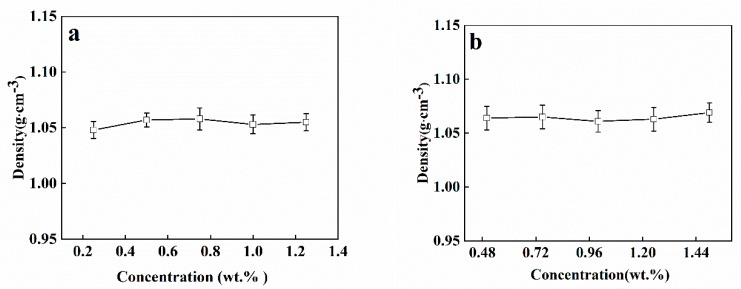
The density of (**a**) SA solutions at concentrations of 0.25, 0.5, 0.75, 1.0 and 1.25 wt.% and (**b**) CaCl_2_/CMC solutions at CMC’s concentrations of 0.5, 0.75, 1.0, 1.25 and 1.5 wt.%.

Figure 4 shows that the concentrations of SA and CaCl_2_/CMC solution have limited impact on the density of the solution. The densities of SA (1.05–1.06 g cm^−3^) and CaCl_2_/CMC (1.06–1.07 g cm^−3^) solution are close to that of water (1.00 g cm^−3^). According to previous researches [31], the density of alginate solution showed minimal impact on the size and shape of Ca-alginate beads formation because the increment degree of alginate solution’s density is only marginal, which is close to that of water. So, we did not make further experiments.

### 3.2. Tuning Mechanical and Permeation Properties by Controlling SF

The *SF* of mm-CaSA-Caps is detailed in Table 1 in relation to the designed experiments. Spherical mm-CaSA-Caps (*SF* < 0.07) were obtained within the shaded area, which conformed with the viscosity of SA solutions < 72.0 mPa·s and the viscosity of CaCl_2_/CMC solutions > 56.7 mPa·s.

**Figure 5:**
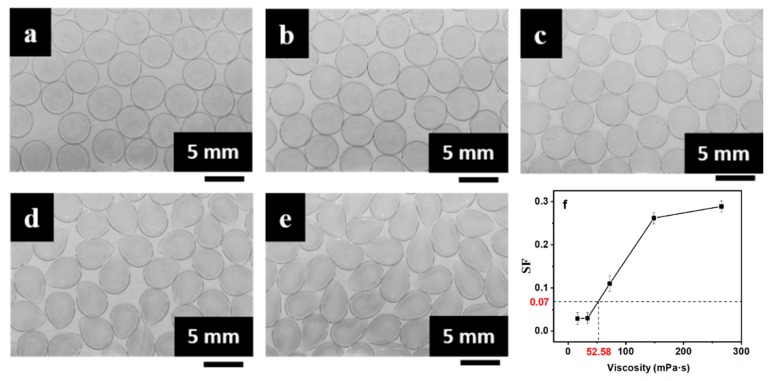
Effect of SA solution viscosity (**a**) 16.2 mPa·s, (**b**) 33.6 mPa·s, (**c**) 72.0 mPa·s, (**d**) 148.5 mPa·s and (**e**) 265.5 mPa·s on (**a**–**e**) morphologies and (**f**) *SF* of the mm-CaSA-Caps when the viscosity of the CaCl_2_/CMC solution is 917.5 mPa·s. The concentrations of SA were 0.25, 0.5, 0.75, 1.0 and 1.25 wt.%. The concentrations of CaCl_2_ was 1.5 wt.%. The concentrations of CMC was 1.5 wt.%.

Images of the mm-CaSA-Caps produced by different viscosities of SA solution with the viscosity of CaCl_2_/CMC solution at 917.5 mPa·s are shown in Figure 5a–e. The mm-CaSA-Caps changed from spherical pearl shapes to teardrop shapes when the viscosity of the SA solution increased from 16.2 to 265.5 mPa·s. Plotting the average *SF* of column data in Table 1 against the viscosity of the SA solution^1^ produced the curve shown in Figure 5f. The *SF* of the mm-CaSA-Caps increased from 0.029 to 0.289. Uniform and spherical capsules could be formed when the viscosity value of the SA solution was less than 52.58 mPa·s.

**Figure 6:**
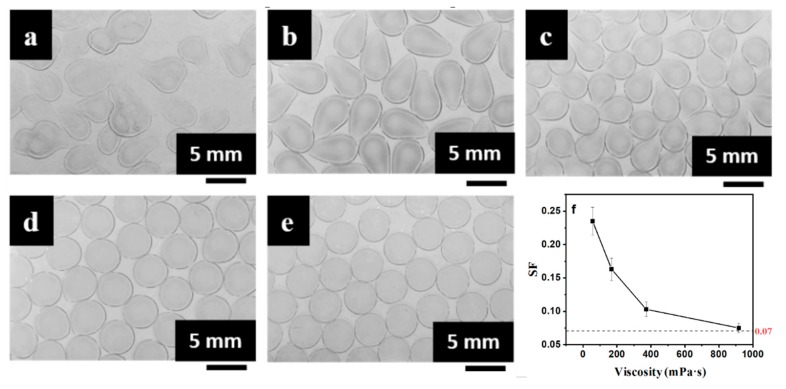
The effect of CaCl_2_/CMC solution viscosity (**a**) 20.0 mPa·s, (**b**) 56.7 mPa·s, (**c**) 168.5 mPa·s, (**d**) 372.0 mPa·s and (**e**) 917.5 mPa·s on morphology and (**f**) *SF* of mm-CaSA-Caps when the viscosity of the SA solution is 72.0 mPa·s. The concentrations of SA were 0.75 wt.%. The concentrations of CaCl_2_ was 1.5 wt.%. The concentrations of CMC were 0.5, 0.75, 1.0, 1.25 and 1.5 wt.%.

Images of the mm-CaSA-Caps produced by different viscosities of CaCl_2_/CMC solution with the SA solution viscosity of 72.0 mPa·s are shown in Figure 6. No mm-CaSA-Caps could be formed when the viscosity of the CaCl_2_/CMC solution was too low, i.e., 20.0 mPa·s, because serious deformation was caused when CaCl_2_/CMC droplets with low viscosity impacted on the surface of the viscous SA solution [22]. With an increase in the viscosity of the CaCl_2_/CMC solution, mm-CaSA-Caps formed. A significant shape variation was observed for the range of viscosity values from 56.7 to 168.5 mPa·s. The CMC as a thickening agent was added into the CaCl_2_ solution to modulate the viscosity and density of the core layer solution to ensure the formation of spherical capsules [8]. The deformation of droplets on impact with the SA solution surface could be minimized and consequently spherical capsules could be formed with low viscosity SA solution and high viscosity CaCl_2_/CMC solution [27,32]. Uniform and spherical mm-CaSA-Caps were formed with further increase in CaCl_2_/CMC viscosity. Taking the average *SF* of column data in Table 1 and plotting it against the viscosity of the CaCl_2_/CMC solution produced the curve shown in Figure 6f. That curve shows that the viscosity of the SA solution must be below 72 mPa·s and the viscosity of the CaCl_2_/CMC solution must be between 56.7 and 917.5 mPa·s to obtain spherical mm-CaSA-Caps with *SF* < 0.07.

**Figure 7:**
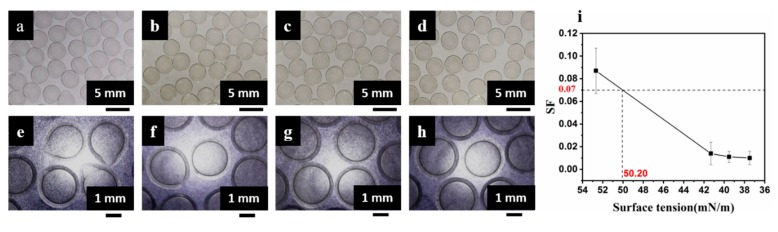
Images of mm-CaSA-Caps prepared with different surface tensions of SA/poloxamer 407 solution (**a**) 47.01 mN/m, (**b**) 41.71 mN/m, (**c**) 39.6 mN/m, (**d**) 37.17 mN/m; (**e**–**h**) are higher magnification images of (**a**–**d**) respectively and (**i**) *SF* of the mm-CaSA-Caps. The concentration of SA was 0.5 wt.%. The concentration of CaCl_2_ was 1.5 wt.%. The concentration of CMC was 1.0 wt.%. The concentrations of poloxamer 407 were 0, 0.01, 0.05 and 0.2 wt.%.

**Figure 8:**
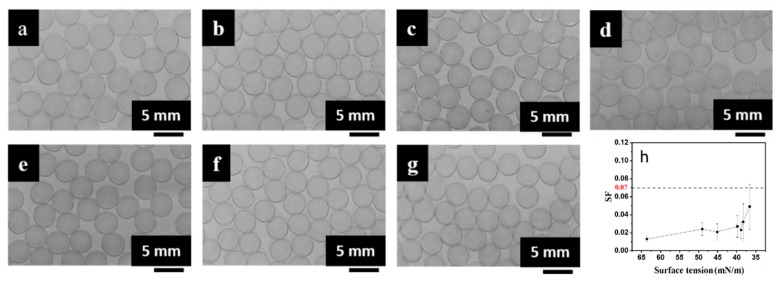
Images of the mm-CaSA-Caps prepared at different surface tensions of CaCl_2_/CMC/Tween 80 solution (**a**) 63.61 mN/m, (**b**) 49.07 mN/m, (**c**) 45.14 mN/m, (**d**) 39.85 mN/m, (**e**) 38.86 mN/m, (**f**) 38.29 mN/m, (**g**) 36.63 mN/m and (**h**) *SF* of the mm-CaSA-Caps. The concentration of SA was 0.5 wt.%. The concentration of CaCl_2_ was 1.5 wt.%. The concentration of CMC was 1.0 wt.%. The concentrations of Tween 80 were 0, 0.015, 0.025, 0.038, 0.075, 0.15 and 0.3 g/L.

The effect of the surface tension of the coagulation bath, i.e., SA/poloxamer 407, on the shape of the mm-CaSA-Caps was investigated. From Figure 7, the mm-CaSA-Caps prepared in 0.5 wt.% SA solution without poloxamer 407 were pear-shaped and had an obvious “tail” appearance, as shown in Figure 7a, e with *SF* = 0.087, which was above 0.07 (Figure 7i). It has been proved that non-spherical shape CaSA beads not only reduced the gel strength, but also resulted in uncontrolled release rate of the encapsulant compared to that of spherical beads [31]. After the addition of a small amount of poloxamer 407, the sphericity of capsules obviously improved, although there were still individual irregular capsules, as shown in Figure 7b,f with *SF* decreased to 0.014 (Figure 7i). The sphericity and uniformity of the capsules improved continuously while the surface tension of SA/poloxamer 407 solution continued to decrease and the *SF* decreased to 0.011. However, the surfactant poloxamer 407 had a limited effect on the surface tension of the SA solution, as shown in Figure 3a. The capsule shape did not obviously change with the increase in the concentration of poloxamer 407 (Figure 7 and Figure 8). It has been proved that the penetration depth of droplets is mainly affected by the viscosity and surface tension of the bath solution [1]. During the production of the CaSA capsules, a surfactant was added to the gelation bath to reduce surface tension, and hence the impact force could be reduced to obtain spherical capsules [31]. Thus, the surfactant addition enhanced penetration and prevented shape deformation of the CaSA capsules. 

The effects of the surface tension of the inner layer solution on the size and shape of the mm-CaSA-Caps is shown in Figure 8a–g. It can be observed that uniform and spherical capsules were obtained at different surface tensions of CaCl_2_/CMC/Tween 80 solution. As shown in Figure 8h, as the surface tension of the solution decreased from 63.41 to 36.63 mN/m, the *SF* of capsules increased but still remained below 0.07 and the *D* of capsules decreased from 4.18 to 3.88 mm. Spherical beads can be obtained with low impact force and high penetration depth, depending mostly on the surface tension of bath solution and the viscosity of droplets [1,31]. The surface tension of droplets had less impact on *SF* but a decrease in the surface tension of droplets was not beneficial to the form of spherical capsules.

**Figure 9:**
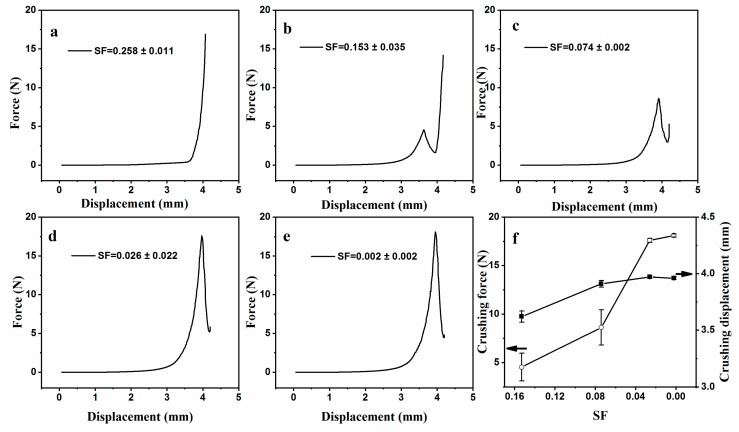
The effect of *SF* on the mechanical properties of mm-CaSA-Caps. The concentrations of CMC were 0.75 (**a**), 1.0 (**b**), 1.15 (**c**), 1.25 (**d**) and 1.5 (**e**) wt.%. The crushing force and the crushing displacement of the Caps with different SF (**f**). The concentration of SA was 0.75 wt.%. The concentration of CaCl_2_ was 1.5 wt.%.

The force–displacement curve of mm-CaSA-Caps with different SF (Figure 9a–e) and the crushing force and the crushing displacement of the Caps (Figure 9f) are shown here. It can be seen that the mm-CaSA-Caps with SF = 0.258 ± 0.011 had no crushing point. They developed uneven capsule walls that functioned as stress concentrators, leading to breakage under a small force when the wall touched the plate of the rheometer and the touching force was beyond the rheometer’s detection limit. With the decrease in *SF*, the crushing point appeared and the crushing force and crushing displacement of the Caps clearly increased. However, the crushing force and crushing displacement appeared to show large errors when the *SF* of the mm-CaSA-Caps was above 0.07, as shown in Figure 9f. It was also evident that the mm-CaSA-Caps with *SF* > 0.07 had unstable mechanical properties. In comparison, the Caps with SF < 0.07 had good mechanical stability. The Caps with SF = 0.002 ± 0.002 had the highest crushing force of 18.1 N and the crushing displacement of 3.96 mm.

**Figure 10:**
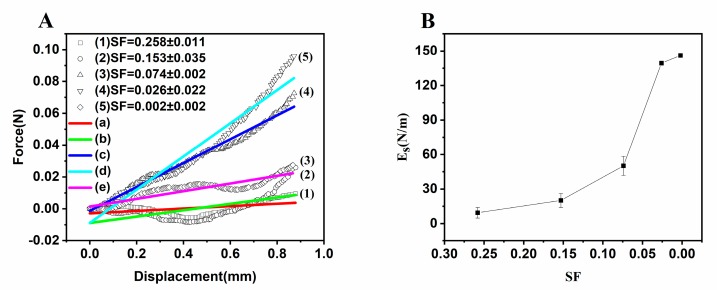
(**A**) Magnification of force–displacement curves of CaSA capsules filled with the different SF for linear regression and (**B**) surface Young’s modulus (E_s_) calculation. The concentrations of CMC were 0.75 (a), 1.0 (b), 1.15 (c), 1.25 (d) and 1.5 (e) wt.%. The concentration of SA was 0.75 wt.%. The concentration of CaCl_2_ was 1.5 wt.%.

Force–displacement curves with the displacement of 0–1 mm were selected and linearly fitted to investigate the elastic deformation and surface elastic modulus (*E_s_*) of mm-CaSA-Caps. As shown in Figure 10A, the slope of the initial force–displacement curve increased when the *SF* of Caps decreased. The calculated *E_s_* of the Caps showed similar results (Figure 10B). The spherical Caps (SF < 0.07) had good resistance to deformation, with the *E_s_* of Caps with SF of 0.002 and 0.026 being 146.1 and 139.4 N/m respectively, whereas the *E_s_* displayed a three-fold decrease of 50.1 N/m when the SF of Caps was 0.074. In general, compared with mm-CaSA-Caps with poor sphericity, Caps with good sphericity had better mechanical stability and higher crushing strength under the action of external force. When capsules had greater rigidity, a larger external force was required to achieve the same deformation. Good mechanical properties guaranteed long term stability for protection of the active ingredients, such as additives, fragrance, pesticides and decorative pearls used in food, home care products, agriculture and cosmetics areas [33].

**Figure 11:**
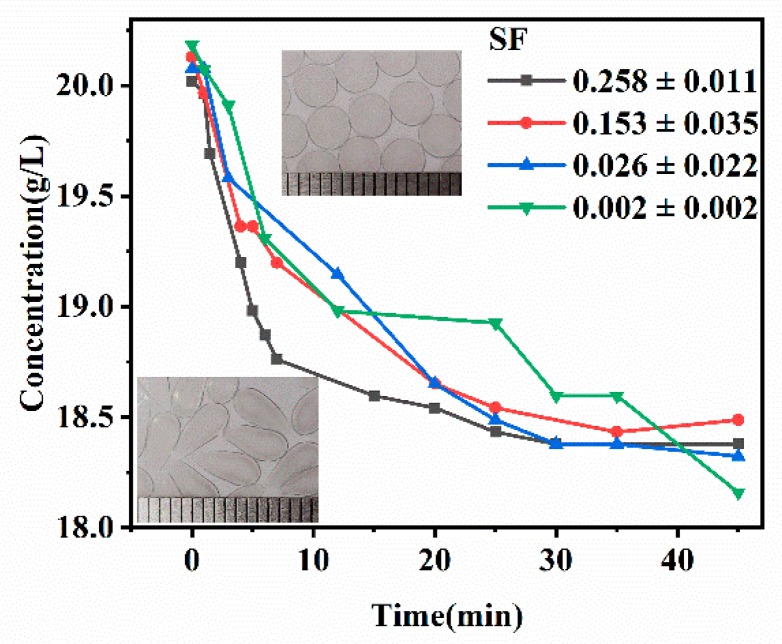
The effect of *SF* on the permeability of mm-CaSA-Caps. The concentrations of CMC were 0.75 (*SF* = 0.258 ± 0.011), 1.0 (*SF* = 0.153 ± 0.035), 1.25 (*SF* = 0.026 ± 0.022) and 1.5 (*SF* = 0.002 ± 0.002) wt.%. The concentration of SA was 0.75 wt.%. The concentration of CaCl_2_ was 1.5 wt.%.

Effect of SF on the permeability of mm-CaSA-Caps was investigated via measurement of the diffusion of glucose from the bulk solution into intra-hollow Caps. As shown in Figure 11, a decrease of glucose concentration produced different behaviors with different SF of mm-CaSA-Caps below and above 0.07. The mm-CaSA-Caps with SF > 0.07 showed a faster initial permeation rate and shorter equilibrium time than observed in the mm-CaSA-Caps with SF < 0.07. Unlike the uniform capsule wall thickness of the spherical Caps, the pear-shaped Caps had thin wall structure at their tip, leading to rapid permeation of glucose. The spherical Caps had a more even permeation rate, conducive to uniform and sustained release of a loaded drug. Furthermore, the millimeter-scale alginate capsules are extensively used to encapsulate cells and immobilize enzymes. It is also important to adjust and control the permeability of solutes across the Ca-alginate capsule membrane [7].

### 3.3. Size Control and Prediction

**Figure 12:**
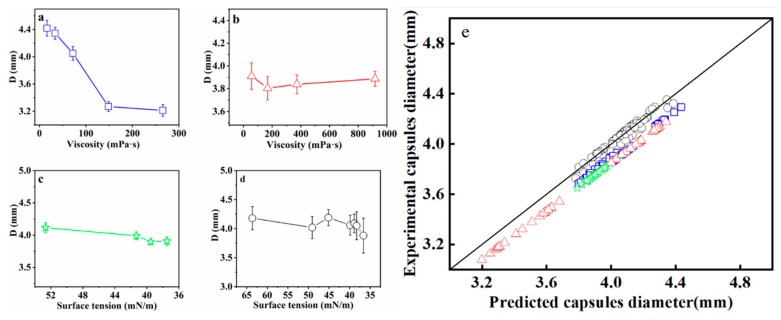
(**a**) Effect of SA solution viscosity on diameter of the mm-CaSA-Caps. The viscosities of the SA solution were 16.2 mPa·s, 33.6 mPa·s, 72.0 mPa·s, 148.5 mPa·s and 265.5 mPa·s when the viscosity of the CaCl_2_/CMC solution was 917.5 mPa·s; (**b**) effect of CaCl_2_/CMC solution viscosity on the diameter of the mm-CaSA-Caps. The viscosities of the CaCl_2_/CMC solution were 20.0 mPa·s, 56.7 mPa·s, 168.5 mPa·s, 372.0 mPa·s and 917.5 mPa·s when the viscosity of the SA solution was 72.0 mPa·s; (**c**) *D* of mm-CaSA-Caps prepared at different surface tensions of SA/poloxamer 407 solution; (**d**) *D* of mm-CaSA-Caps prepared at different surface tensions of CaCl_2_/CMC/Tween 80 solution and (**e**) validation of the capsule diameter prediction model.

**Figure 13:**
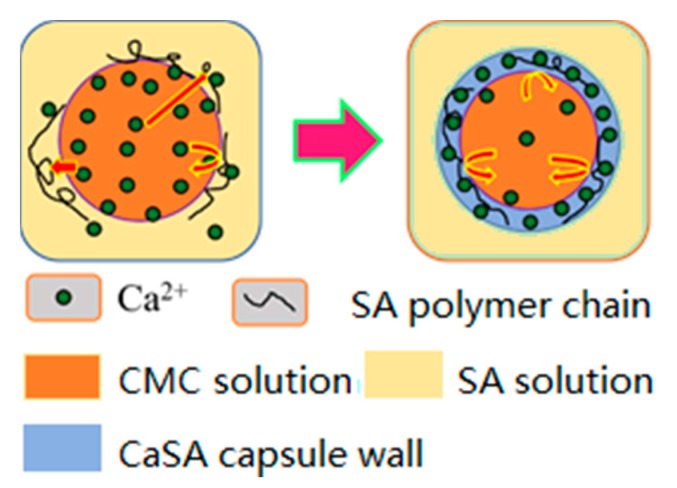
Formation of the wall of mm-CaSA-Caps and its barrier mechanism.

The results in Table 2 show that the outer layer SA solution and the inner layer CaCl_2_/CMC solution had different levels of effect on the diameter of capsules. As the viscosity of SA solution increased from 16.2 to 265.5 mPa·s, the diameter of capsules decreased significantly under the different CaCl_2_/CMC concentrations. Taking the average *D* of column data in Table 2 and plotting it against the viscosity of SA solution produces the curve shown in Figure 12a. The average diameter of mm-CaSA-Caps decreased 27.3%, i.e., from 4.418 to 3.213 mm. As the CaCl_2_/CMC solution was dripped into the SA solution, the chelate reaction occurred quickly on the interface of CaCl_2_/CMC and SA. The speed and degree of the reaction were controlled by the bidirectional diffusion of SA molecules and Ca^2+^, mainly by the Ca^2+^ diffusion rate. Since the number of SA molecules per unit volume increased at high SA concentrations, the number of binding sites of –COO^−^ for Ca^2+^ also increased, leading to higher degrees of crosslinking than those formed with lower SA concentrations. The crosslinking structure thus formed could prevent the Ca^2+^ diffusion, while the high viscosity of the SA solution as the external phase also decreased the diffusion rate of Ca^2+^, as illustrated in Figure 13. Therefore, the capsule diameter no longer increased in a short time [6]. The diameters of capsules formed with high SA concentrations were lower than those formed with low SA concentrations.

From Table 2, the diameter of the mm-CaSA-Caps decreased as the viscosity of the CaCl_2_/CMC solution increased when the viscosity of the SA solution was ≤ 33.6 mPa·s. However, no apparent trend was observed when the CaCl_2_/CMC concentration increased with the viscosity of the SA solution ≥ 72.0 mPa·s. It has been reported that the hydroxyl groups in CMC have the potential to form hydrogen bonding with alginate carboxyl groups to enhance the mechanical stability of capsules [12,34]. Thus, when the viscosity of SA was low, the crosslinking density of CaSA was low during the initial reaction period of the CaCl_2_/CMC drop entering the SA base, facilitating easier bidirectional diffusion of CMC and SA molecular in the interface. As the viscosities of CaCl_2_/CMC and SA solution were both low, the diameter of capsules was high because of the easier polymer chain diffusion and hydrogen bonding formation. With the increase in viscosity of the CaCl_2_/CMC solution, even the diffusion of the CMC chain became easier, as the CMC and SA molecules would have more hydrogen bond connection, increasing the interface viscosity and preventing further diffusion of CMC molecular chains. Therefore, the capsule diameters decreased with the increase in the viscosity of CaCl_2_/CMC solution under the low viscosity of SA solution. When the viscosity of the SA solution increased, the molecular diffusion was mainly affected by the high crosslinking density of CaSA and the high viscosity of the SA solution. Effects on the viscosity of CaCl_2_/CMC solution were not apparent. The diameter of the mm-CaSA-Caps no longer changed. Taking the average *D* of row data in Table 2 and plotting it against the viscosity of the CaCl_2_/CMC solution produced the curve shown in Figure 12b, demonstrating that no apparent trend was observed when the CaCl_2_/CMC concentration increased, as reported by others [11,35], the reason being that the concentration of Ca^2+^ was not changed in the CaCl_2_/CMC solution, further evidence that the diameter of the mm-CaSA-Caps was mainly controlled by the Ca^2+^ diffusion.

The effects of surface tension of the coagulation bath (SA/poloxamer 407) on the size of mm-CaSA-Caps were investigated. The capsules diameters decreased a little with the decrease of the surface tension of SA/poloxamer 407 solution, as shown in Figure 12c. It had been reported that decreasing the surface tension of a gelation bath was beneficial to decreasing capsule deformation but had no apparent influence on the size of capsules [31] and beads [1]. The effects of the surface tension of the inner layer solution on the size of mm-CaSA-Caps are shown in Figure 12d. The surface tension of droplets had a greater impact on the diameter of the capsules [36], which decreased as the surface tension of CaCl_2_/CMC solution decreased.

**Figure 14:**
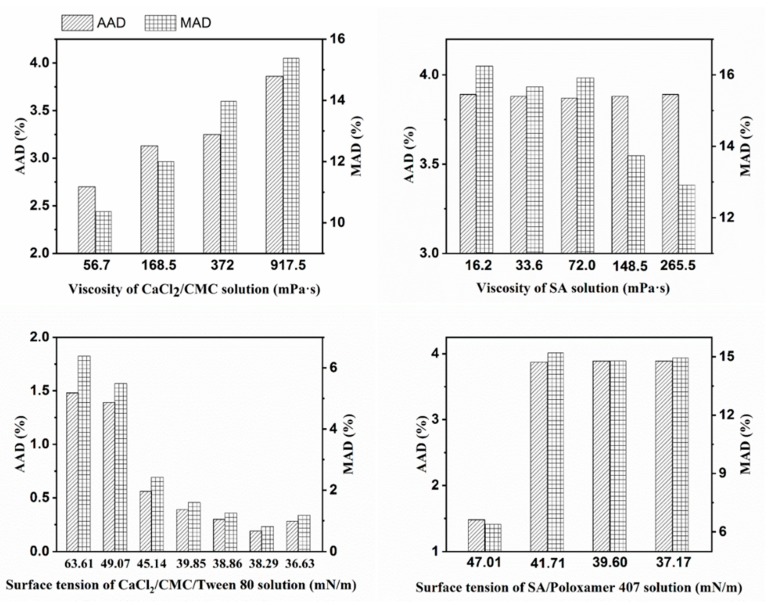
Error analysis of the diameter of mm-CaSA-Caps.

The diameter of mm-CaSA-Caps was predicted by Equation (2). The predicted diameter was verified with experimental data to evaluate the accuracy and reliability of the capsule’s diameter prediction model for mm-CaSA-Caps. As presented in Figure 12e, the predicted diameter was in good agreement with the experimental data. Error analysis of the capsule’s diameter predicted by the model (Figure 14) shows that the AAD and MAD were < 4% and 15%, respectively. This result demonstrated that the modified prediction model could be used to predict the diameter of mm-CaSA-Caps within reasonable deviation.

## 4. Conclusions

Millimeter-scale calcium-alginate capsules (mm-CaSA-Caps) were produced by the extrusion dripping method based on the ionotropic gelation principle. The viscosity and surface tension of SA solution and CaCl_2_/CMC solution were considered as major factors for tuning the shape of mm-CaSA-Caps. The results indicated that viscosities of SA solution and CaCl_2_/CMC solution were both critical factors for the preparation of spherical mm-CaSA-Caps. Deformed capsules were formed at high SA viscosity or low CaCl_2_/CMC solution viscosity, due to impact forces that distorted the shape of CaCl_2_/CMC droplets when they hit the surface of the viscous SA solution. A small amount of surfactant in the CaCl_2_/CMC solution was helpful for decreasing the *SF* of the mm-CaSA-Caps. For the diameter of mm-CaSA-Caps, the ranking of influential parameters is the viscosity of SA solution>surface tension of CaCl_2_/CMC solution > surface tension of SA solution > viscosity of the CaCl_2_/CMC solution. The diameter of capsules was predicted based on the modified Tate’s law with an absolute deviation of less than 4%. The SF of mm-CaSA-Caps had a marked impact on the mechanical properties and permeability of mm-CaSA-Caps. Spherical Caps had superior mechanical stability, higher crushing strength and higher steady-state permeation rate, findings that have broad application prospects in the food and medical fields.

## Figures and Tables

**Table 1:** Effects of viscosity of SA and CaCl_2_/CMC solutions on the sphericity factor (*SF*) of mm-CaSA-Caps.

Viscosity of CaCl_2_/CMC (mPa·s)	Viscosity of SA (mPa·s)				
	16.2	33.6	72.0	148.5	265.5
20.0	/	/	/	/	/
56.7	0.067 ± 0.026	0.086 ± 0.029	0.258 ± 0.011	0.394 ± 0.016	0.371 ± 0.024
168.5	0.032 ± 0.019	0.018 ± 0.011	0.153 ± 0.035	0.289 ± 0.012	0.323 ± 0.007
372.0	0.009 ± 0.006	0.006 ± 0.004	0.026 ± 0.022	0.230 ± 0.014	0.242 ± 0.011
917.5	0.009 ± 0.006	0.008 ± 0.008	0.002 ± 0.002	0.136 ± 0.009	0.219 ± 0.009

**Table 2:** Effects of viscosities of SA and CaCl_2_/CMC solution on *D* (mm) of the mm-CaSA-Caps.

Viscosity of CaCl_2_/CMC (mPa·s)	Viscosity of SA (mPa·s)				
	16.2	33.6	72.0	148.5	265.5
20.0	/	/	/	/	/
56.7	4.769 ± 0.137	4.757 ± 0.129	3.951 ± 0.087	2.940 ± 0.087	3.131 ± 0.150
168.5	4.355 ± 0.176	4.243 ± 0.109	3.847 ± 0.115	3.336 ± 0.074	3.239 ± 0.042
372.0	4.271 ± 0.071	4.221 ± 0.037	4.323 ± 0.176	3.208 ± 0.067	3.167 ± 0.065
917.5	4.278 ± 0.081	4.170 ± 0.072	4.076 ± 0.027	3.597 ± 0.054	3.313 ± 0.091
